# Interdisciplinary evaluation of a robot physically collaborating with workers

**DOI:** 10.1371/journal.pone.0291410

**Published:** 2023-10-11

**Authors:** Andrea Cherubini, Benjamin Navarro, Robin Passama, Sonny Tarbouriech, Shirley A. Elprama, An Jacobs, Susanne Niehaus, Sascha Wischniewski, Freek J. Tönis, Pim L. Siahaya, Giorgia Chini, Tiwana Varrecchia, Alberto Ranavolo

**Affiliations:** 1 LIRMM, Univ Montpellier, CNRS, Montpellier, France; 2 IMEC-SMIT-Vrije Universiteit Brussel, Brussels, Belgium; 3 Federal Institute of Occupational Safety and Health, Dortmund, Germany; 4 Hankamp Gears, Enschede, The Netherlands; 5 INAIL, DiMEILA, Monte Porzio Catone (RM), Italy; Polytechnic University of Marche: Universita Politecnica delle Marche, ITALY

## Abstract

Collaborative Robots—CoBots—are emerging as a promising technological aid for workers. To date, most CoBots merely share their workspace or collaborate without contact, with their human partners. We claim that robots would be much more beneficial if they physically collaborated with the worker, on high payload tasks. To move high payloads, while remaining safe, the robot should use two or more lightweight arms. In this work, we address the following question: to what extent can robots help workers in physical human-robot collaboration tasks? To find an answer, we have gathered an interdisciplinary group, spanning from an industrial end user to cognitive ergonomists, and including biomechanicians and roboticists. We drew inspiration from an industrial process realized repetitively by workers of the SME HANKAMP (Netherlands). Eleven participants replicated the process, without and with the help of a robot. During the task, we monitored the participants’ biomechanical activity. After the task, the participants completed a survey with usability and acceptability measures; seven workers of the SME completed the same survey. The results of our research are the following. First, by applying–for the first time in collaborative robotics–Potvin’s method, we show that the robot substantially reduces the participants’ muscular effort. Second: we design and present an unprecedented method for measuring the robot reliability and reproducibility in collaborative scenarios. Third: by correlating the worker’s effort with the power measured by the robot, we show that the two agents act in energetic synergy. Fourth: the participant’s increasing level of experience with robots shifts his/her focus from the robot’s overall functionality towards finer expectations. Last but not least: workers and participants are willing to work with the robot and think it is useful.

## Introduction

In today’s factories, many operators face work-related health problems. In particular, musculoskeletal disorders account for a significant proportion of sickness leaves [1]. Among manual material handling (MMH) activities, several physical risk factors have been identified, including handling low load at high frequency and heavy lifting. Collaborative robots (CoBots, [2–4]) are a promising solution for improving the workers’ ergonomics, without introducing new risks caused by the robots themselves. In fact, robot and worker contribute to the task, with what each does best. For instance, the robot’s accuracy, repeatability and strength can complement human intelligence [5]. While robots are often present in assembly-line production facilities such as car manufacturers [6–10], they mostly address lightweight tasks, and are seldom in physical contact with humans [11]. Referring to the taxonomy defined in [12], most current day robots are only sharing their workspace or contactlessly collaborating with their human partners.

Instead, in our research, we design and assess a more ambitious scenario, where the robot should physically collaborate with humans on a high payload operation. To design and evaluate such innovative technology, we have gathered an interdisciplinary group [13], spanning from an industrial end user to cognitive ergonomists, and including biomechanicians and roboticists.

Consider a manual industrial process, which could be modified by the addition of a robot physically collaborating with the workers. The scientific questions addressed in this article are: how would the addition of this robot affect the industrial process and the workers and their physical well-being? To what extent will the workers adopt/accept this technology? Can we verify that the robot operation is repeatable/reliable for the end user standards?

To address the above questions, we have applied the following method. With the help of the SME HANKAMP (Netherlands), we have reproduced one of their industrial processes in our Laboratory. Participants had to replicate the process, without and with the help of the robot. During the task, we monitored the participants’ biomechanical activity. After the task, the participants completed a survey with usability and acceptability measures; 7 workers of the SME had completed the same survey. The experiments mentioned above are shown in the video available at: https://gite.lirmm.fr/humar/research-projects/sophia_dataset_example/-/blob/main/videos/rgbd_dataset_overview.mp4

The contributions of our work with respect to the state of art are the following.

- For the first time, Potvin technique [14, 15], commonly used in ergonomics to quantify physical effort in repetitive tasks, is applied to assess a collaborative robot.
- We show that, during a collaborative activity, the worker’s muscular effort is correlated to the power measured by the robot, implying that the two agents act in energetic synergy, and that in the near future, the robot could perform the biomechanical risk assessment, while helping the worker.
- We design and present an unprecedented method for measuring robot reliability and reproducibility in collaborative scenarios.
- Using well-known tools for usability and acceptability assessment [16, 17], we show that the participant’s level of experience with robots shifts his/her focus from the robot overall functionality towards more fine-grained aspects (e.g., adaptability and conformity with his/her expectations), and that both industrial workers and participants appear willing to work with the robot and think it is useful.

Beyond these contributions, our work shows how a multidisciplinary approach can help in the holistic evaluation of a collaborative human-robot manufacturing cell.

In the following paragraphs, for each of these aspects, we briefly overview the state of art and we detail the implications of our findings.

Robots are a viable option for lowering biomechanical risk in MMH activities [18, 19]. Indeed, they can physically assist workers to improve their motion and biomechanical performance, which can be measured via wearable sensors, data-model fusion and machine learning techniques [20–26]. Although there exist many hypotheses and scientific evidence regarding the beneficial effects of Human-robot collaboration (HRC) technologies, research on human side effects lacks [27–29]. In this work, we perform an instrumental-based assessment of the biomechanical risk during the execution of an occupational task, executed without and with the robot. In particular, using sEMG, we investigate the upper limb behaviors as input to the Potvin technique [14, 15], The Potvin technique–used by occupational health and safety technicians, ergonomists, occupational physicians, biomedical engineers and movement scientists to quantify physical effort in repetitive tasks–has never been applied to assess collaborative robots.

In the present article, we also show that the activity of the human biceps muscle and the power measured by the robot are correlated. This is a very promising result. First, it shows that worker and robot act in energetic synergy. Second, it could allow robots to perform biomechanical risk assessments without requiring workers to wear devices–as is the case today [30].

To move high payloads, while remaining safe, the robot should use two or more lightweight arms. This implies the need for redundant controllers, which often yield non-repeatable trajectories [31]. Additional causes of uncertainty are: the presence of a human in the loop, and the need for sensor (vision and force) feedback loops, to operate in a dynamic environment. While there exist many studies on determining the repeatability of industrial manipulators [32–38], most of them focus on single arms controlled at the joint level, and none of them concurrently addresses the three aspects mentioned above (redundancy, human physical collaboration, and sensor-based control). In this work, we propose a new method for assessing the repeatability of robots, and we show that our controller is capable of 0.05 rad median joint precision on the target use case. Another original result is the evaluation of the reliability of a collaborative robot in an industrial use case. We show that our framework has 97% reliability on the target use case. These results are very promising, in view of the integration of collaborative robots in future workplaces.

In this work, we also investigated whether the robot can be handled with effectiveness, efficiency, and satisfaction, so that users can achieve certain goals in a particular context. In other words, we investigated the robot’s usability [39]. Usability has become increasingly important in the field of industrial robotics [40, 41]. Usability can be assessed by surveys such as well-known and frequently applied System Usability Scale (SUS) [16, 42]. Additionally, the ISO standard 9241–110:2019 [43] provides seven general design principles for the system design: Suitability for the task, Self-Descriptiveness, Controllability, Conformity to User Expectations, Error Tolerance, Suitability for Individualization and Learnability [39, 43]. We analyzed all seven principles within this study. The 2020 configuration also includes User Engagement, while Individualization is encompassed in Controllability [44]. However, the used questionnaire was developed with the previous configuration. Prior experience can influence the user’s attitude towards robots, including usability, trust and acceptance [45]. We found that the level of experience in working with robots and the familiarity with them influences the way users rate Human-Robot Interaction principles. While people with low experience seem to focus on the mere suitability for the task, people familiar with robots give more importance to individualization aspects. In sum, greater experience seems to shift the focus from the robot overall functionality, indicated by suitability for the task and error tolerance, towards more fine-grained aspects, such as adaptability and conformity with the user’s expectations.

We also investigated the acceptance of robots based on measures originating from one of the most well-known and frequently used models [17, 46, 47]. We assessed acceptance prior to implementation, and we found that perspective robot users are willing to employ them and think they are useful.

## Materials and methods

### Use case

The objectives of this study are: understanding if and how a robot can impact the human’s physical effort in a high payload industrial use case, and assessing the robot’s reliability, usability and acceptability.

**Fig 1:**
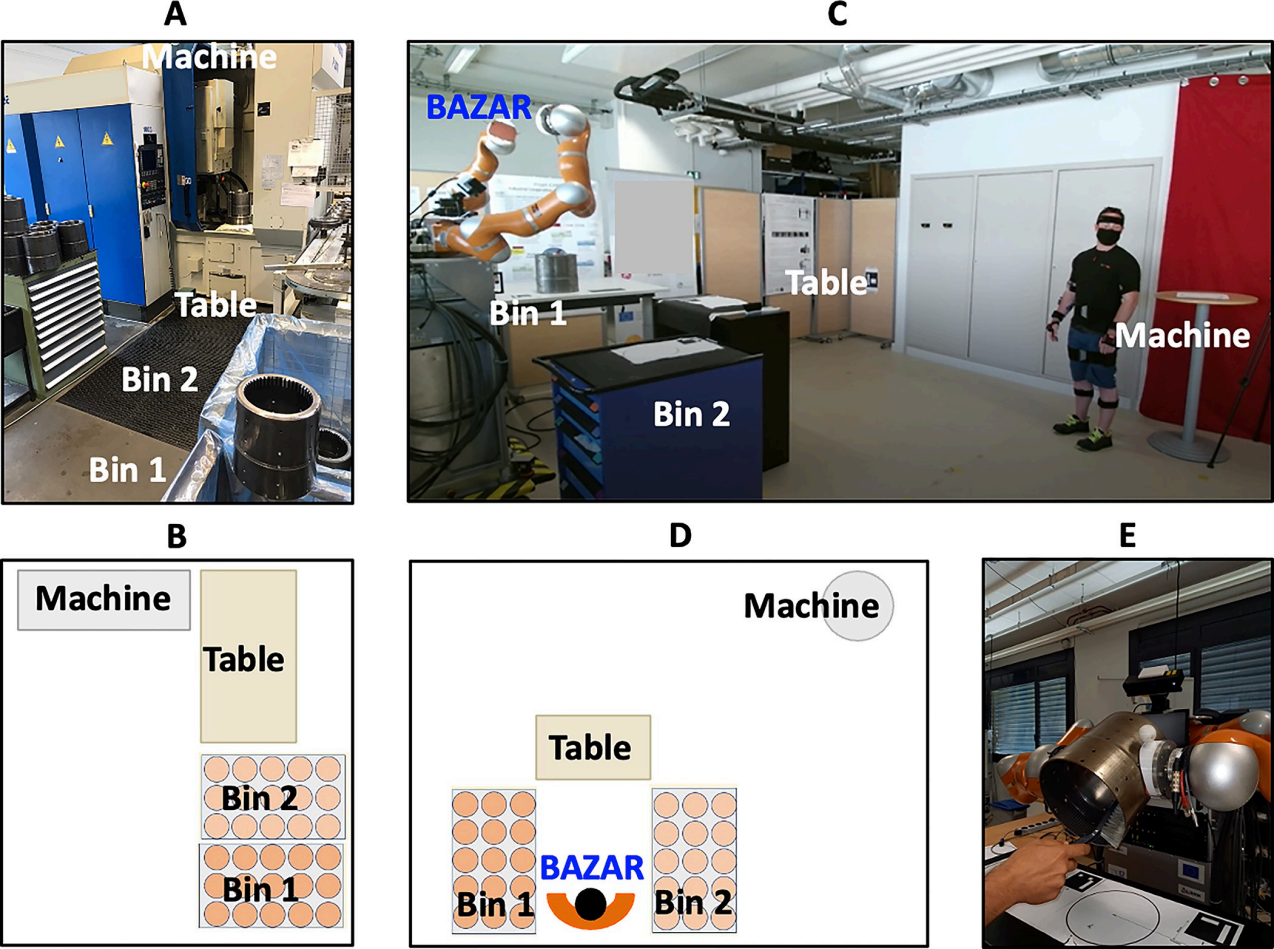
The gear shaping scenario and its components. (**A**) Real scenario at SME HANKAMP with (**B**) layout. (**C**) Mock-up scenario at Université de Montpellier, with (**D**) layout and (**E**) detail of the deburring phase in collaboration with BAZAR. The detail of the gear shaping process is given in Fig 2.

**Fig 2:**
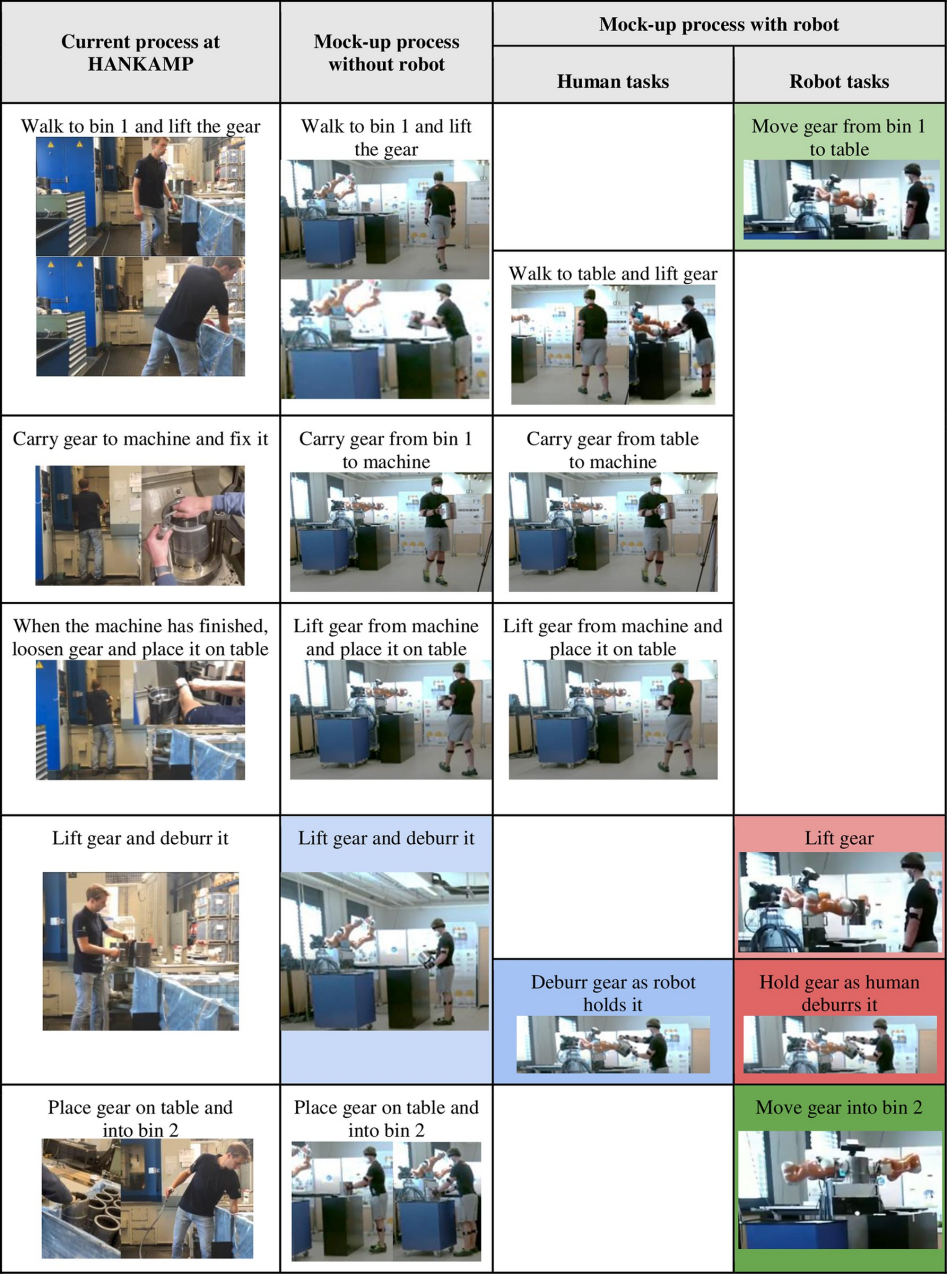
Comparison between the current process and our experiment. The table reports all tasks (realized by either human or robot) in: the current industrial process, the laboratory mock-up process without the robot, and the laboratory mock-up process with the robot.

We drew inspiration from “gear shaping”, an industrial process realized repetitively by workers of the SME HANKAMP (Netherlands). Fig 1 illustrates the gear shaping scenario, and Fig 2 details the gear shaping process. The process concerns a component of a gear produced at HANKAMP, which may weigh between 0, 1 and 20 kg, and which will be denoted gear in the following. The workers must move the gear in a pre-defined order, between four places in the factory, and deburr it with a brush and with air pressure. These places are two bins (denoted bin 1 and bin 2 in the following), P300 Gear Shaper machine (denoted machine in the following), and a table. At Université de Montpellier, we designed and analyzed a mock-up of the HANKAMP SME gear shaping process (see Fig 1C–1E)), including dual arm mobile robot BAZAR [48], referred to as BAZAR in the following. Eleven participants (5 females and 6 males; age: 27.73±5.99 years; body mass index: 23.06±3.93 kg/m2) took part in the study. Six of them identified themselves as students, and the 5 others as workers. The experiments were carried out in accordance with the Helsinki Declaration and authorized by the Université de Montpellier EuroMov’s Laboratory Ethics Committee (Protocol number IRB-EM 2103A –see https://drive.google.com/file/d/1FyIrhEVnEczKjeAVytyVDWWhx01WlXcr/view?usp=sharing). All participants, after being introduced to data privacy issues and voluntariness of the participation, gave their written consent to participate in the study. The individuals in this manuscript have given written informed consent (as outlined in PLOS consent form) to publish these case details. Participants’ exclusion criteria included inability to give informed written consent, history of musculoskeletal disorders, limb or trunk surgery, orthopedic or neurological diseases, disorders of the vestibular system, visual impairments or back pain, current pregnancy, current pharmacological treatment, and obesity.

Each participant completed the task six times: three times without (denoted woB) and three times with (wB) BAZAR. We randomly ordered the two conditions (w and wo BAZAR), for each participant. Before the participant performed either condition for the first time, we showed him/her a video of the task, and let him/her execute it once, without recording. Furthermore, to obtain the isometric maximal voluntary contractions (MVCs), we asked each participant to perform a specific exercise twice with each muscle [49–55].

**Fig 3:**
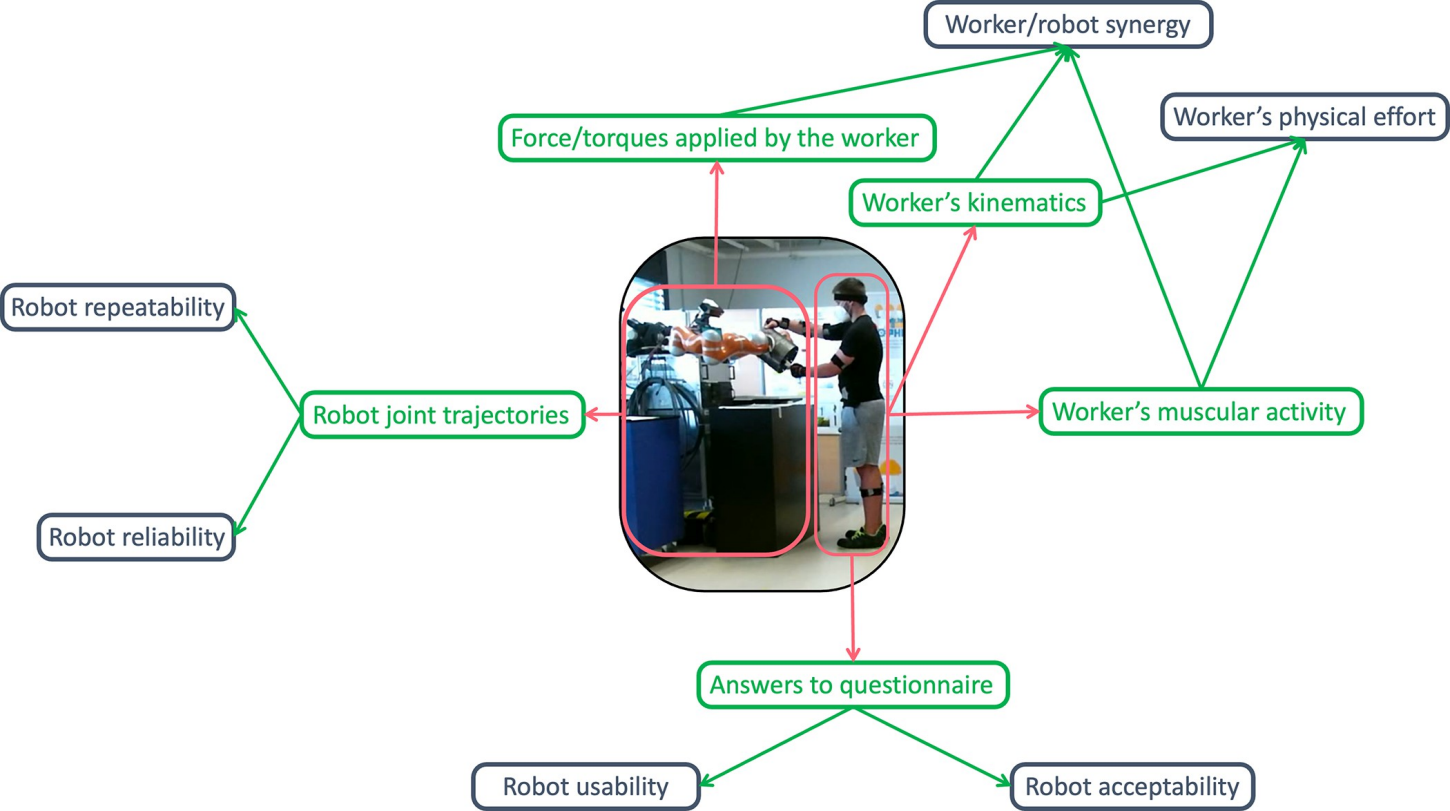
Mindmap of the experimental procedure used in this work. **Red**: Involved agents (robot and worker). **Green**: data measured on the agents **Blue**: features of the human-robot collaboration derived from the measured data.

Fig 3 illustrates a mindmap outlining how we used measures on the robot and participants (workers) to derive relevant features of this human-robot collaboration.

We took the gear shaping process at HANKAMP Gears SME (see the leftmost column of Fig 2) as the basis for this experiment, since this task is repetitive and often performed in poor ergonomic positions. This process includes manual lifting and assembly of heavy (up to 20 kg) gears.

The robot should perform the lift/place tasks to reduce the risks of injuries. To alleviate the workers physical strain, the robot must:

1. load heavy (up to 20 kg) gears, while compensating the weight;
2. include assistive functions to help the worker place the gear in the correct position;
3. move among the four places, while being able to reliably perceive them;
4. accommodate different ranges of gears with various sizes through adaptable grippers.

We only focused on requirements 1–3, by designing two (one per arm) customized grippers, to fit one gear size. We used a robot with two arms (BAZAR) since high payloads (above 15 kg) are incompatible with most current day CoBot arms [56, 57].

The mock-up scenario (Fig 1C–1E) consisted of: 4 tables, 1 gear (5.3 kg), 1 cleaning brush and BAZAR. Fig 2 shows (left to right) the current industrial process, the laboratory mock-up process woB, and the laboratory mock-up process wB. In designing the latter (third and fourth columns), BAZAR does the most tiring subtasks (first column). These tasks are lifting/placing the gear from/into bins (which require bending), and deburring the gear (which requires holding the gear in the air with one hand). To avoid humans from lifting the gear from bin 1, the robot moves it from bin 1 to the table (light green task in Fig 2), which is closer to the humans’ starting position and at a more ergonomic height than the bin. The same applies for the last placing task (dark green): the robot moves the gear from the table to bin 2. The human deburring task requires BAZAR to lift the gear from the table (light red) and to hold it in the air while the human deburrs it (dark red). BAZAR is purposely not used for tasks involving the machine, since these require fine manipulation capabilities (for fixing and loosening bolts). BAZAR’s usefulness in lifting/placing the gear from/into bins is evident, since it avoids human bending and reduces the walking distance by 1 m per task. Instead, the contribution to the deburring phase is more difficult to assess, and it will be studied in the biomechanical analysis. Furthermore, this task is also innovative, since it falls in the physical collaboration category [12].

### Measuring physical effort

To evaluate the participants’ biomechanical effort, we relied on a Xsens (Xsens, Enschede, The Netherlands) MVN Link suit with inertial measurement units (IMU) and on Wi-Fi surface electromyographs (FreeEMG300 System, BTS, Milan, Italy) at 1000 Hz, as sEMG. We used Xsens MVN Analyze software (v2018.0.0) to record IMU data at 60 Hz, and 16-channel.

The IMU and sEMG data were acquired simultaneously, synchronizing the two devices. MVN Awinda system motion analysis system includes a protocol comprising 17 IMUs placed all over the body to measure the orientation of body segments [50], hence the whole-body kinematics. We acquired sEMG signals via bipolar Ag/AgCl surface electrodes (diameter 2 cm, H124SG Kendall ARBO, Tyco healthcare, Neustadt/Donau, Germany), placed with electroconductive gel over the muscle belly, in the direction of the muscle fibers, and with 2 cm between the electrodes’ centers. This configuration complies with European recommendations for surface electromyography [49–51] and with the atlas of muscle innervation zones. We placed bipolar electrodes on the flexors and extensors of six joints: anterior deltoideus (AD) and posterior deltoideus (PD) on the shoulder, biceps brachii caput longum (BBCL) and triceps brachii caput longum (TBCL) on the elbow, and finally flexor carpi radialis (FCR) and extensor carpi radialis (ECR) on the wrist. We chose the sensors’ type and configuration so as not to interfere with the workers’ motor strategy for active task participation. The sensors have wireless communication and are miniaturized (light weight and small). Additionally, we placed sEMG below the clothing and IMUs in elastic bands away from body joints. Besides, the participants practiced to become familiar with the procedure and equipment, before the recording session. For all these reasons, participants experienced no restrictions while moving.

To measure physical effort during the deburring task (two light blue cells in Fig 2), we analyzed the participants’ sEMG via the Potvin approach [14, 15], which is adopted by ergonomists and engineers to quantify efforts in repetitive tasks. To assess repetitive occupational tasks, Potvin provides–for each muscle–a Tolerance Limit Value (TLV) of the duty cycle percentage (%DC) in function of the maximal voluntary contraction percentage (%MVC).

We identified the deburring task via the vertical velocities of the feet and hands, acquired with Xsens. We conducted a first segmentation from the feet velocity signals, to distinguish the phases where the subject moved from one place to the other, and within that, a finer segmentation, using the hand velocity signals, to identify the start/end of load lifting. Then, the envelope of each sEMG signal was extracted using full-wave rectification and low-pass filtering with 4^th^ order Butterworth filter at 5 Hz [26].

According to the Potvin method, the equation to compute the TLVs is:

%MVC=(100%)(−0.143ln(DC/(100%))+0.066)

where %MVC is the percent of maximum strength or effort of the hand, elbow or shoulder and DC is the duty cycle, expressed as a percentage of the total work cycle. The duty cycle is the percent of time–over a work cycle or over a certain time period–force is applied. The TLV fatigue curve can be used to compute acceptable percent duty cycle for a given force (%MVC) or an acceptable %MVC for a given percent duty cycle. The TLV applies to duty cycles within the range of 0.5% to 90%. The TLV is intended for cyclic work normally performed for 2 or more hours per day. If a worker does multiple tasks that are each more than 2 hours long, none of the tasks should exceed the TLV. Static exertions of the hand, elbow or shoulder should not exceed 20 minutes. The recommended limits apply specifically to the upper limb.

The statistical analyses were performed using SPSS 20.0 (IBM SPSS). For each participant and each condition (wB and woB), the data of the three trials were averaged. Preliminarily, we applied the Shapiro-Wilk normality test, to check whether the data had a normal distribution. A two-way repeated measures ANOVA was performed to determine if there was any significant effect on the Potvin results, among the %MVC and between wB and woB. Wherever the ANOVA test had shown a main effect, we carried out the post hoc analysis, with Bonferroni’s correction. We considered p values lower than 0.05 as statistically significant.

### Measuring the robot performance

BAZAR [48] is a dual arm mobile robot, equipped with two 7-DOF Kuka LWR4 arms attached to a Neobotix MPO-700 omnidirectional mobile base. A Microsoft Kinect camera is mounted on BAZAR’s head, and an ATI Mini 45 force/torque sensor is mounted on each of its wrists. We equipped each wrist with a 3D printed concave part, designed to fit the gear, and denoted ‘hand’ in the paper. We used the camera to detect the spots on the tables for lifting/placing the gear, and the force sensors both to regulate the grasp and to make BAZAR comply with the participants’ manual guidance.

BAZAR’s framework is programmed in C++ within RKCL [58], a hierarchical quadratic programming library that applies task prioritization. The framework is executed on a computer with an Intel(R) Xeon(R) E5-2620 v3 CPU running Linux with the PREEMPT-RT patch. RKCL is available at https://gite.lirmm.fr/rkcl/rkcl-core

To localize the three places (two bins and table) where the gear should be lifted or placed, we rely on QR codes. We use the Microsoft Kinect V2 RGB-D camera in BAZAR’s head and the ArUco Library [59], which provides real-time marker-based 3D pose estimation.

For trajectory generation, we used the Reflexxes Motion Library [60] with: maximum translational velocity = 0.5 m/s, maximum rotational velocity = 0.5 rad/s, maximum translational acceleration = 0.2 m/s^2^ and maximum rotational acceleration = 0.2 rad/s^2^.

To make the robot compliant, we rely on admittance control [61], by deforming the trajectories of each hand via a spring-damper model. The deformation will depend on pre-tuned damping (B) and stiffness (K) gains. By varying the gains, we designed three admittance control modes: *pose*, *force*, and *damping control*, with the parameters given in Table 1.

**Table 1:** Parameters of the three admittance control modes. Damping (B) and Stiffness (K) gains along the translational and rotational degrees of freedom for pose, damping and force control modes.

Degrees of freedom	Translations		Rotations	
Parameter	B (in Ns/m)	K (in N/m)	B (in Ns/rad)	K (in N/rad)
**Pose control**	150	250	25	40
**Damping control**	150	0	25	0
**Force control**	1000	0	500	0

In this experiment, BAZAR had to execute three sub-tasks (see Fig 2):

1. Move the gear from bin 1 to table. Actions: position arms (before grasp), grasp gear, lift gear, turn, place gear, position arms (to release gear).
2. Lift gear and hold it as human deburrs it. Actions: position arms (before grasp), grasp gear, lift gear, move gear according to the human applied force and torque.
3. Move gear into bin 2. Actions: turn, place gear, position arms (to release gear).

These rely on the following actions:

a1) Position arms: for this action, since the hands are not constrained to one another (i.e., they are not holding anything) we control them independently with *pose control*.
a2) Grasp gear: we move the hands independently using *force control* to grasp the gear.
a3) Turn base: BAZAR’s base rotates until the robot sees the ArUco marker placed on the bins or table.
a4) Move gear: for this action, we use the cooperative task representation [62]. Instead of controlling each arm independently, we consider them as a unique entity and define their motion via an absolute task (which controls the pose of the gear in the workspace), and a relative task (which regulates the pose of one hand with respect to the other). When there is no human interaction, the absolute task is *pose controlled*, whereas during ‘brushing’ we control the gear vertical translation and tilt angle with *damping control*. By compensating gravity [58], only the human forces guide the robot. This allows him/her to move the gear with minimal effort.

We recorded data from the robot at 20 Hz. To measure joint repeatability, we proceeded as follows.

- We computed the median duration (across all experiments) of each of the three tasks, to obtain: 30.65 s, 34.91 s, 23.13 s.
- We resampled the joint trajectories of the 3 tasks, so that each trajectory lasts as long as the median duration.
- For each joint trajectory, at each iteration we calculate median and interquartile range across all experiments.
- For each joint, we calculate median and interquartile range across all experiments, of the distance between the joint value and the median at the given iteration.

The above operations were realized in Python 3.6, with the support of the following libraries: math, pandas, numpy, matplotlib and scipy.

### Relating the worker activity with the robot power

In **Measuring physical effort**, we have explained how we measured BBCL %MVC.

To measure the robot power during brushing, we computed the scalar product between the wrench (force and torque) and the velocity vectors, both applied in the robot’s absolute task frame.

Then, for each participant and trial, we aligned (starting from the brushing phase) the envelope of BBCL muscle and the curves of robot power during the interaction phase, resampled to 200 points and extracted the mean values [26]. The Pearson test was used to investigate the correlation between mean values of BBCL and robot power. We considered p values lower than 0.05 as statistically significant.

During brushing, we control the gear vertical translation and tilt angle with damping control, while blocking the 4 other gear degrees of freedom (see **Measuring the robot performance**)

The velocity along these 4 degrees of freedom is null; therefore only two of the six components of the wrench are used to calculate the robot power. This is a limitation caused by our design choice of the collaborative control scheme: the robot cannot measure the power exchanged via the 4 other components of the wrench applied by the human. A solution to this could consist in letting the human control all 6 degrees of freedom (with damping control) during brushing. Nevertheless, the correlation observed between BBCL activity and robot power leads us to think that participants quickly adapt to the damping control, and apply wrenches only along the two useful degrees of freedom (vertical translation and tilt angle).

### Measuring usability and acceptability

The usability and acceptance survey was completed by all 11 participants and by 7 workers at HANKAMP.

One set of questions measured the participants’ previous experiences (2 items) and their perception of mass media (5 items). The usability of the system was measured with the well-known System Usability Scale (SUS, [16]). As a base for the assessment of the usability of this kind of system, we used a short and adapted version of the IsoMetrics for robots (7 items), where each item refers to one of the dialogue principles from ISO 9241. As demographic variables, we asked the participants information about sex, year of birth, seniority, highest obtained degree and profession.

Acceptability was measured using the concepts *Intention to use* (3 items), *Effort expectancy* (2 items), *Attitude* (3 items), *Performance expectancy* (3 items), *Facilitating Conditions* (2 items) and *Social Influence* (2 items) (concepts originally from well-known and frequently used work [17]). We adapted the items to include robots, based on [63].

To create a value for prior experience, we accumulated the two questions on working experience and familiarity with robots for each participant. The same applied to answers on the system usability scale (SUS). The questionnaire has been applied in a variety of research projects and industrial evaluations and yields high reliability as well as item validity. Due to the low number of participants, we used descriptive statistics to analyze the ranking of the interaction principles as well as the usability survey. Following t-tests were performed to determine if there was any significant effect of prior experience on the interaction principles and perception of usability aspects.

We checked Cronbach’s alpha values of the acceptance measures, which is an indicator (0 to 1) whether multiple questions are measuring the same underlying concept. All measures showed an acceptable internal reliability (0.7 or higher, see [64]) for the workers, but for the participants some measures showed questionable to poor reliability, namely for Performance Expectancy (ɑ = 0.568), Facilitating Conditions (ɑ = 0.426) and Attitude (ɑ = 0.520). We calculated a single score for each concept for both groups, by adding all items per concept and calculating their mean. The statistical analyses were performed using SPSS version 28 (IBM SPSS). P-values lower than 0.05 were considered statistically significant.

## Results

### Results on human biomechanical effort

Let us first comment on the instrumental-based assessment of the biomechanical risk of the deburring task woB and wB (blue and red cells in Fig 2). The human motion duration during this task was similar woB (12.79±0.79 s) and wB (12.10±1.66 s). Hence, the robot’s aid does not reduce the cycle time.

**Fig 4:**
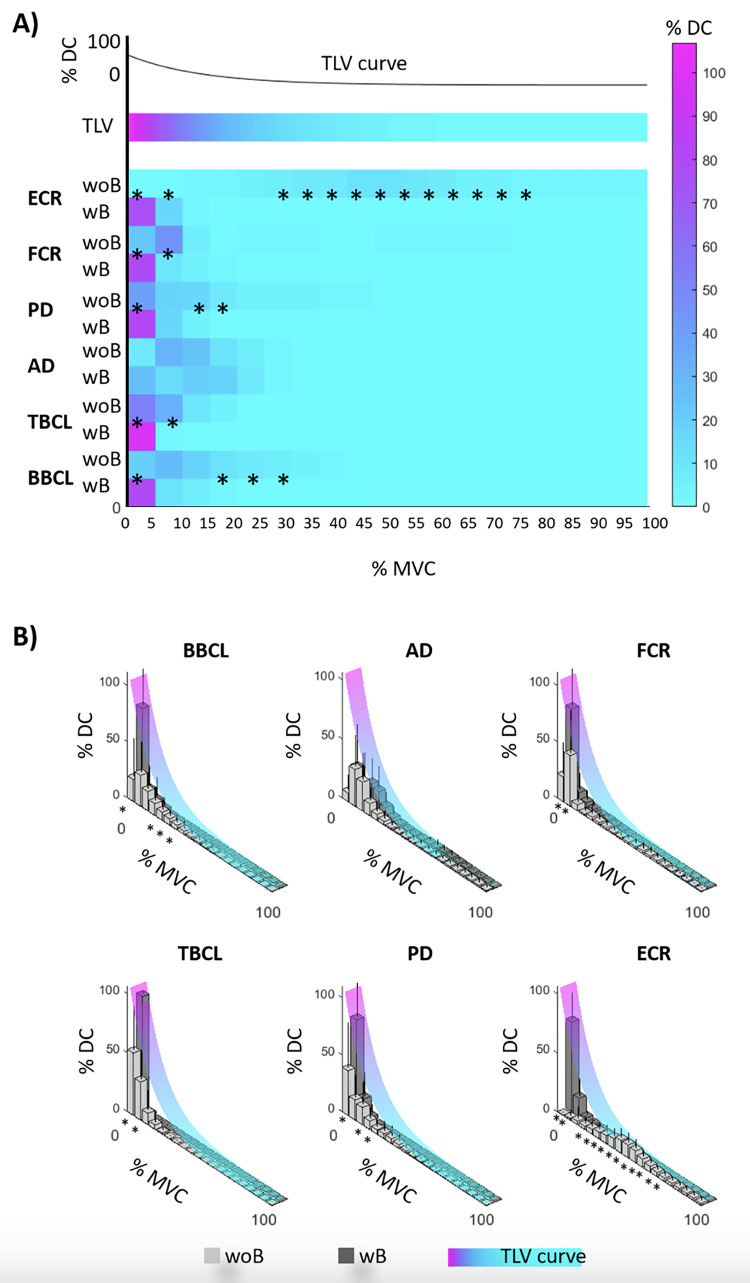
Potvin results with (wB) and without (woB) CoBot. **(A)** Tolerance Limit Value (TLV) curves and mean duty cycle (%DC) along participants, in function of the maximal voluntary contraction (%MVC) of six right upper arm muscles (BBCL: biceps brachii caput longum; TBCL: triceps brachii caput longum; AD: anterior deltoideus; PD: posterior deltoideus; FCR: flexor carpi radialis; ECR: extensor carpi radialis). **(B)** TLV curves with mean and SD of the %DC among all participants in function of %MVC (light grey: woB, dark grey: wB). * Significant differences.

Yet, the Potvin approach shows that the robot reduces muscular fatigue during task execution. Fig 4 shows the Potvin Map for both conditions: wB and woB. Fig 4A shows the TLV curves and the mean among all participants of %DC in function of %MVC, for six muscles of the right upper arm. Fig 4B shows the TLV curves and the mean and SD among all participants of %DC in function of %MVC for the six muscles, woB (light grey) and wB (dark grey).

The two-way repeated ANOVA measures show a significant effect of the interaction %MVC*task condition (wB and woB) on Potvin (F = 11.673, df = 19, p<0.001). In other words, BAZAR modifies muscle fatigue during task execution. Particularly, post hoc analysis (see Fig 4A) shows that %DC wB is significantly higher than woB within 0–5% MVC for biceps brachii caput longum (BBCL, p<0.001), triceps brachii caput longum (TBCL, p = 0.003), posterior deltoideus (PD, p = 0.005), flexor carpi radialis (FCR, p<0.001) and extensor carpi radialis (ECR, p<0.001). This implies that–when working wB–participants maintain very low muscle activation, in the 0–5% MVC range, for almost the entire task execution. Furthermore, post hoc analysis shows that %DC wB is significantly lower than woB:

- 5–10% MVC for TBCL (p = 0.002), FCR (p = 0.029) and ECR (p = 0.019),
- 10–15% MVC for PD (p = 0.048),
- 15–20% MVC for BBCL (p = 0.045) and PD (p = 0.024),
- 20–25% MVC for BBCL (p = 0.022),
- 25–30% MVC for BBCL (p = 0.023) and ECR(p = 0.029),

• 30–35, 35–40, 40–45, 45–50, 50–55, 55–60, 60–65, 65–70, 70–75, 75–80% MVC for ECR (respectively p = 0.006, 0.001, 0.001, 0.001, 0.002, 0.002, 0.002, 0.002, 0.011, 0.038).

We found no significant differences between wB and woB, for anterior deltoideus (AD).

**Fig 5:**
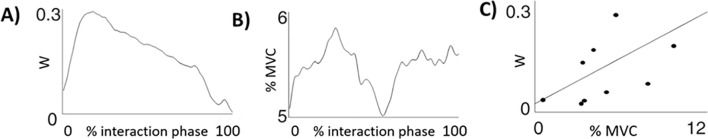
**(A)** Mean curve of BAZAR power (in W) among all subjects. (**B**) Mean curve of Biceps brachii caput longum (BBCL) %MVC among all subjects. (**C**) Correlation among mean values of BAZAR power and BBCL %MVC.

We obtained another original result (see Fig 5) by comparing the %MVC with the power measured by BAZAR at its wrists. During the interaction phase, the mean of BBCL among all participants was 5.56±5.04% MVC and the power measured by BAZAR was 0.146±0.145 W. The Pearson test showed a positive correlation between these two values, with p = 0.002 and r = 0.822: the higher the power transferred to BAZAR, the greater the biomechanical effort. This result confirms that the robot interacts in energetic synergy with the participants, and that in the future the robot force/torque sensors might be used (instead of sEMG) to assess the worker biomechanical risk during the task.

### Results on robot control

An important contribution of our work is the assessment of the reliability and repeatability of a collaborative robot in an industrial use case. These assessments rely on an important number of repetitions: 3 per participant (i.e., 33 in total), all novice (i.e., without previous robot experience).

We obtained a very high reliability (97% success rate; 1/33 fails). These results are unique in the context of such complex experiments, involving humans, multiple integrated robots (two arms, the camera pan-tilt unit and the mobile base) and sensor-based control. Furthermore, the lone failure was due to an issue with the FLIR PTU-E46 pan-tilt unit self-calibration: a 0° tilt angle command made the camera look downwards, instead of straight ahead. Identifying the angle offset and compensating it in the robot controller could fix this issue. It should also be noted that the pan-tilt actuation is never used, since the camera always maintains a fixed position with respect to BAZAR’s chest. Thanks to visual feedback and to force control, BAZAR could grasp the gear even when its position was not precisely known. In practice, we observed that it could tolerate offsets up to 2 cm from the pre-defined position. This error is tolerable in the process, since the human is still in charge of the tasks requiring the most accuracy, e.g., fixing/loosening the gear onto/from the machine.

**Fig 6:**
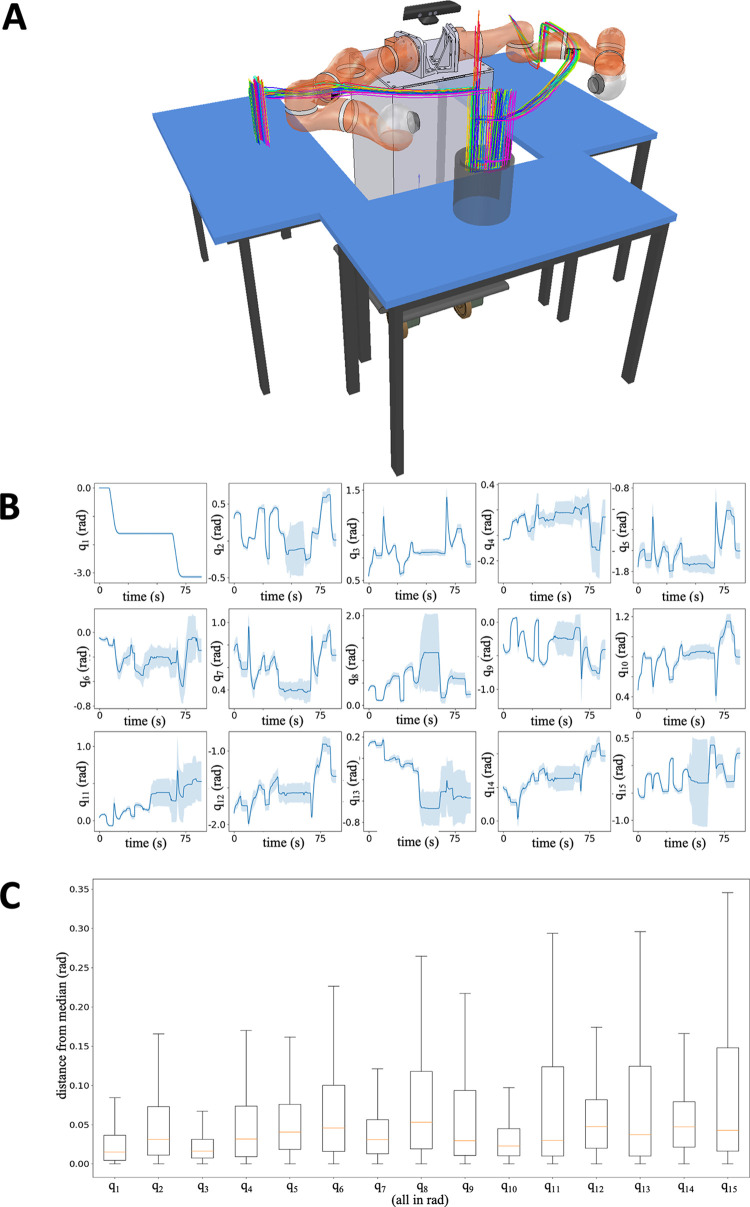
**(A)** Qualitative rendering of BAZAR operation with the trajectories of the gear center for each of the 33 experiments. (**B**) Joint angle trajectories q_1_…q_15_ (in rad) across all experiments with median (dark blue) +/- range (light blue). Top to bottom and left to right: heading of the base, left arm joints and right arm joints. (**C**) Boxplots of the precisions (distances from the median) of the 15 joint angles, in the same order as in (**B)**.

Besides reliability, a second fundamental criterion (rarely considered in the literature) is path repeatability. The robot will have predictable–hence, safe–behavior from the human viewpoint, if its motion is constrained within a thin envelope around some nominal trajectory. This is not trivial for a sensor-controlled robot, since its trajectories depend on a sensor-based feedback loop, and therefore are affected by noise and environment variability (including human interaction). Fig 6A provides a qualitative outline of the trajectories of the gear for each of the 33 experiments. To obtain it, we imported the model of BAZAR and the setup in CoppeliaSim simulator [65]. We then plotted the (X,Y,Z) coordinates of the absolute task pose for all experiments.

BAZAR’s controller [58] is redundant with respect to the task, since it uses 15 degrees of freedom (heading of the base and 7 joints per arm) to control the 6 dimensional pose of the gear. To verify BAZAR’s repeatability, we proceeded as follows. First, we studied separately the three robot tasks (see Fig 2): move gear from bin 1 to table, lift gear and hold it as the human deburrs it, and move gear into bin 2. The Shapiro-Wilk test showed that the robot data was not normally distributed (p < 0.05) so we hereby give the median +/- range. A first, promising result is the low variability of the duration of these three tasks: 30.65±0.90 s, 34.91±1.10 s, 23.13±0.75s. Within each of the tasks, we analyzed the 15 robot joint trajectories. These are plotted in Fig 6B with median (dark blue) +/- range (light blue). It should be noted that–as expected–the highest variability occurs during the interaction phase (roughly from 31 s to 66 s). Fig 6C shows the boxplots of the precisions (distances from the median) of the 15 joint angles. The figure shows that for all 15 distances, the median distance is lower than 0.05 rad. This is a very promising result, considering that the interaction phase is also considered in the calculation.

The duration of the entire process (including human motion) was: 51.93±4.15 s without BAZAR, and 91.94±4.26 s with BAZAR. HANKAMP’s current process requires approximately 57 s: 13 s for the first two tasks (Walk to bin 1 and lift gear + Carry gear to machine and fix it), 35 s for the third and fourth (Loosen gear and place it on table + lift gear and deburr it) and 9 s for the last task (Place gear on table and into bin 2). While we did not reproduce some parts of the process (e.g., the fixing and loosening), the time cycles are definitely acceptable by HANKAMP, whose current daily production capacity is 104 products per 16 hours (i.e., one gear every 554 s).

### Results on usability and acceptability

Besides the 11 participants of the robot experiment, 7 workers at SME HANKAMP, who perform manually (i.e., without robot) gear shaping, filled out subjective measures on usability and acceptance. The only difference was that the workers viewed a 3D simulation of their prospective workplace with a robot. We hereby refer to these separate groups (N = 11 + 7 = 18) as “workers” and “participants”.

The workers’ *Overall experience with robots* (3.6±0.9), and the *robot’s expected usability* (3.5±1.1) were fairly high. The participants gave *Error tolerance* (assessed by the statement “Operator errors will not lead to serious consequences”) the lowest value (3.4±1.1). This is in accordance with the participants, who also least expected the robot to be error tolerant. Workers from HANKAMP mostly expected *Suitability for the task* with a mean 4.4±0.5, followed by *Adaptability* (4.3±0.5). Again, this tendency can be found in the participant data, where these two principles were among the top three.

**Fig 7:**
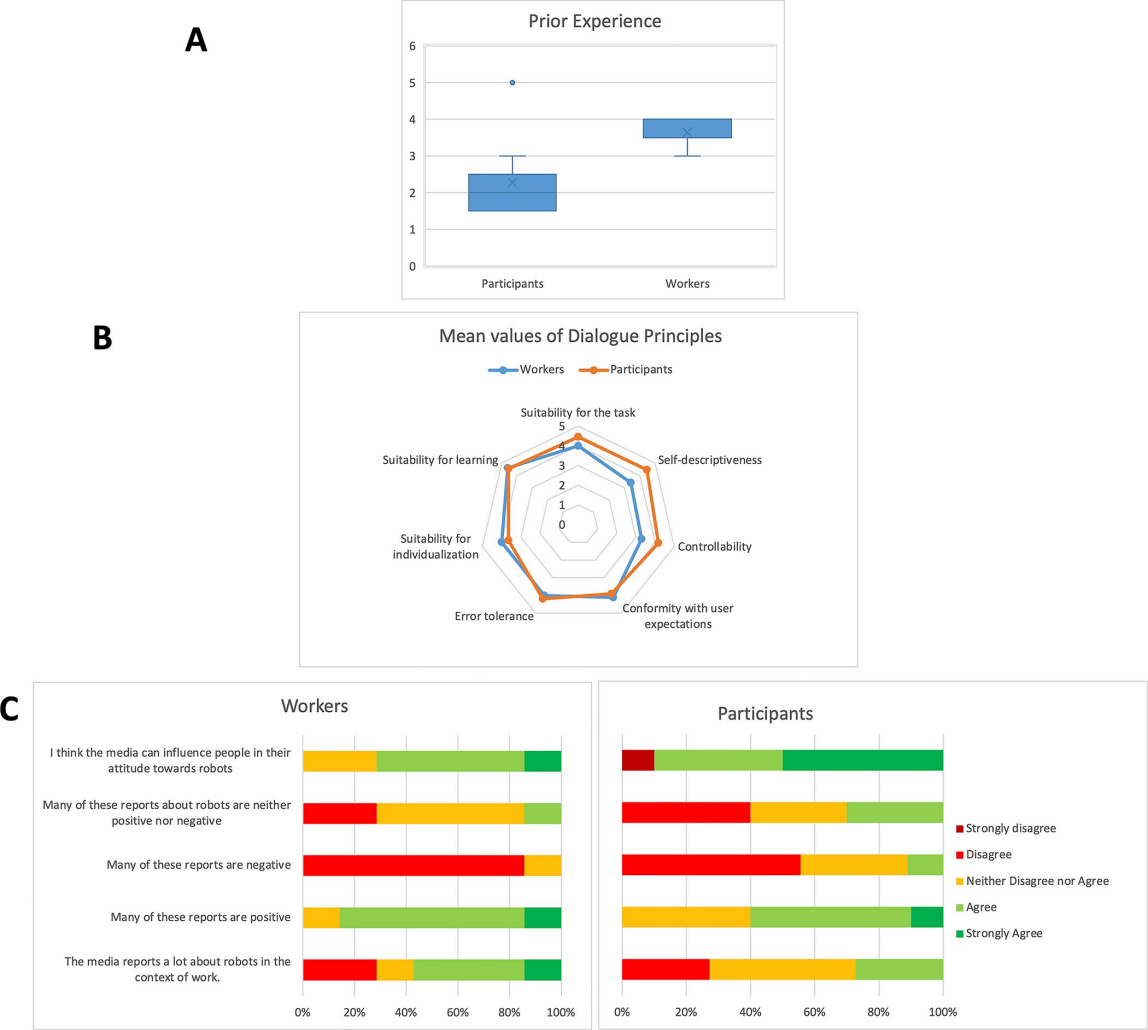
Results on usability aspects; comparison between participants and workers. **(A)** Level of prior experience is higher for workers. **(B)** Differences regarding Dialogue principles: participants value most Suitability for the task, while workers most expect Adaptability and Ease of Learning. **(C)** Similar perception of mass-media reportage: reports are mostly positive and the media can influence people in their attitude towards robots.

Additionally for the robot experiments, the participants’ *prior experience* and *familiarity* with robots was assessed, via statements”I have a lot of experience working with robots” and”I have often seen robots in real life” on a 5-point scale from strongly disagree to strongly agree. The calculated *overall experience* with robots was low (2.3±1.0). Fig 7A shows the difference in experience between workers and participants. To evaluate the *impact of mass media*, the survey focused on media reports about robots in the workplace. Overall, participants agreed more to the statement that news media reports were positive than negative (similar to the responses of workers, see Fig 7C). We also asked the participants’ whether the media can influence people in their attitude toward robots; the score was very high (4.2±1.2).

Regarding the *System’s Usability*, the score is fairly high (4.0±0.6). An inverted item, regarding the consistency of the system, obtained the highest score, followed by statements about the ease of learning and complexity. This indicates that participants found the system very understandable, and expect to learn using it without much training. Another measure concerned the expected usability of robots for work settings in general. Here, participants most expected *Adaptability* (4.6±0.7), followed by *Suitability for the task* (4.5±0.7) and *Ease of Learning* (4.5±0.9). Participants least expected the system to be error tolerant (3.6±1.0). We also asked them to rank the seven dialogue principles [45] from most important (1) to least important (7), to infer which aspect of robot interaction is of most interest. A comparison between responses from workers and participants is shown in Fig 7B.”The robot has to support the working task”–a statement that represents the principle of suitability–was ranked as most important by more than half the participants (2.5±1.8). It was followed by ease of learning and error tolerance, which fits the data on general expectations regarding robots at work. The least important principle was *Conformity with user expectations* assessed by”Workers will always know what every function of the robot can be used for” (5.6±2.2). The 4 more experienced participants (3.75±0.8) compared to the 7 less experienced ones (4.43±0.2) rated this statement significantly more important, *t*(9) = 1.1, *p* < 0.01. There was also a significant difference regarding error tolerance (t(9) = 0.9, p < 0.05) and ease of learning (t(9) = 0.5, p < 0.05), with both ranked more important by experienced participants. There was no significant effect for the rest of the dialogue principles. Only one participant did not respond to all statements. All dialogue principles and corresponding mean values are presented in Table 2.

**Table 2:** Mean values of dialogue principles and acceptance measures.

	Participants			Workers		
	N	M	SD	N	M	SD
The robot has to support the working task	11	4.45	0.69	7	4.04	0.51
The handling of the robot has to be easy to learn	11	4.45	0.93	7	3.43	0.54
The specific functions of the robot have to be self-explaining	11	4.18	1.08	7	3.29	1.01
Workers will be able to control the actions of the robot at any time	10	3.90	0.88	7	4.07	0.57
Workers will always know what every functions of the robot can be used for	11	4.18	0.98	7	4.03	0.62
Worker errors will not lead to serious consequences	11	3.64	1.03	7	4.04	1.08
Workers will be able to adapt the robot to their own needs and abilities	11	4.55	0.69	7	4.57	0.48
Intention to use	11	4.45	0.56	7	4.43	0.46
Attitudes	11	4.18	0.54	7	4.29	0.40
Performance Expectancy	11	4.39	0.59	7	4.19	0.74
Effort Expectancy	11	4.23	0.75	7	3.71	0.81
Social Influence	11	3.32	0.81	7	3.71	0.64
Facilitating Conditions	11	2.86	0.74	7	2.21	0.70

**Fig 8:**
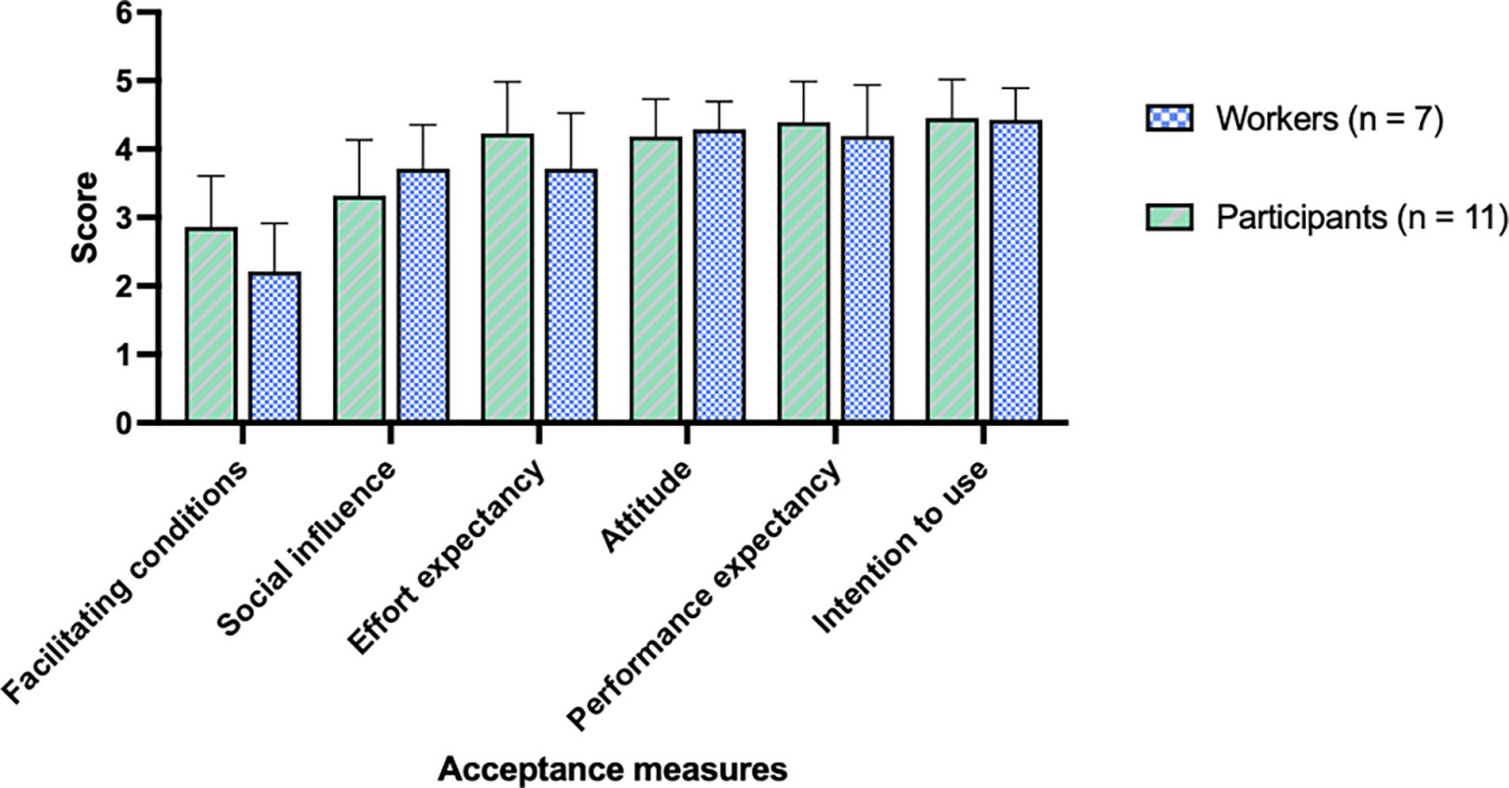
Acceptance measures for workers and participants.

Fig 8 shows the acceptance measures for workers and participants. We only discuss the means and SD of most relevant concepts. Both participants (4.5±0.6) and workers (4.4±0.5) are (very) willing to use robots. For *Attitude* and *Performance expectancy*, we found a similar pattern for both groups, with means between 4.2 and 4.4. This suggests that participants and workers have a positive attitude towards robots and think they are useful.

*Effort expectancy–*or how easy one thinks it is to work with robots—was slightly higher for the participants (4.2±0.8) compared to the workers (3.7±0.8). The workers only saw a video of a simulated CoBot, but there were already two CoBots and six traditional industrial robots in the company when the survey was collected. In contrast, the participants performed a real collaborative task with BAZAR.

### Dataset

These experiments contributed to the production of a dataset, publicly available at **https://gite.lirmm.fr/humar/research-projects/sophia_dataset_open** which contains the data used to reach the conclusions drawn in the manuscript and required to replicate the reported study findings in their entirety. All potentially identifying patient information has been fully anonymized. We expect this dataset to serve as a useful benchmark for researchers interested in HRC worldwide.

## Discussion

This article presents an interdisciplinary joint effort for assessing the impact of a dual arm CoBot in an industrial use case. We successfully managed to reproduce the target process (HANKAMP gear shaping) with the integration of BAZAR to help workers in the most tiring subtasks. These subtasks are lifting/placing the gear from/into bins, and deburring the gear. The robot’s usefulness in lifting/placing the gear from/into bins is evident, since it avoids human bending and reduces the walking distance. Instead, the contribution to the deburring phase is more difficult to assess, and was studied in the biomechanical analysis. Our other objectives were: to evaluate the repeatability and reliability of the robot operation and the participants’ subjective data on robot usability and acceptability.

From a biomechanics perspective, this work has led to two important methodological contributions. First, it is the first work—to the best of our knowledge–which applies Potvin method to quantify the worker’s effort, in a collaborative robot scenario. Second, we show that the worker’s muscular efforts can be correlated to the power measured by the robot. Our findings suggest that using a robot to perform the specific MMH activity reduces the workers’ physical effort as compared to performing the identical task woB. There is a reduction in upper limb muscle activation (Fig 4) for each considered muscle except for AD. Indeed, using the robot, the muscle activation is very small, in the 0–5% of MVC, for all or almost all brushing phases (Fig 4). This result also suggests that the use of a robot cancels the risk of biomechanical overload, as assessed by one of the traditional risk assessment methods listed in part 3 of the ISO 11228. The reduced exposure to biomechanical risks implies better health conditions and a reduced likelihood of the occurrence of a work-related disease in the upper limb. The positive correlation between sEMG data and power measured by the robot is also very promising. In the future, the robot could perform the biomechanical risk assessment of HRC tasks, without having the worker wear cumbersome sensing technologies.

From the robotics viewpoint, the major contribution of our work is the design of a novel method (described in **Measuring the robot performance)** for assessing the reliability and repeatability of a collaborative robot in an industrial use case. To date, and to the best of our knowledge, there exists no such method in the robotics community. In terms of robotics integration, we showed that the robot could meet three among the four industrial requirements: load heavy parts, assist the worker in placing the gear in the correct position and move among the four places. Reliability (97%), Cycle time (92 s) and repeatability (0.05 rad joint precision) all meet the gear shaping process needs. To date, BAZAR is not capable of meeting the fourth requirement, i.e., accommodating different ranges of gears through adaptable grippers. Yet, this is an open problem for the whole robotics community. While soft grippers [66, 67] including suction cups [68, 69] combined with deep machine learning [70] may adjust to shape diversity, these technologies are not capable, to date, of addressing high payloads such as those at issue.

Let us now briefly discuss the subjective evaluation on usability and acceptability.

The results of media-related opinion indicate an overall more positive attitude towards robots. Without any robot experience, individuals might use second-hand opinions as those reported in mass media. However, this did not influence the usability, acceptance, or expectations. The absence of this effect may be explained by the small sample size. Working with the robot was perceived as easy and uncomplex, even for participants which were inexperienced and unfamiliar with robots. This could mean that the system is self-descriptive, so participants know what the robot functions are for. Since there were significant differences between experienced and inexperienced participants in ranking some dialogue principles, it seems that the level of familiarity with these technologies changes the importance of certain interaction principles and usability aspects.

The implementation of robots should always be accompanied by a thorough explanation of the robot’s functions and, if possible, a self-explaining design to ensure good usability. Additionally, the results show that the level of experience with robotic systems has an impact on ranking of usability aspects. Therefore, an ongoing evaluation of the participant’s needs and expectations regarding the system they are working with is crucial to maintain optimal interaction. Moreover, the comparison between results from the laboratory and in the field show that the method used here realistically captures cognitive ergonomics in both scenarios. Although the robot supports humans by reducing the walking distance and by lifting the gear, it is good to learn that the participants and workers also perceive it as useful.

Acceptance of robots can be encouraged by involving workers in the implementation process by e.g. discussing where robots could be useful. Rogers [71] argues that trialability is very important for technology adoption. By involving users, new ideas are more likely to be adopted. Indeed, [72] found that more involved people also had a more positive attitude towards technological innovation. The number of participants involved in this research (n = 18) was low. Ideally, technology acceptance research needs a large number of respondents, due to the nature of the required statistical analysis. Nonetheless, it is very important to include workers since their feedback can increase acceptance.

This research has shown a potential robot end user such as SME HANKAMP that a robot like BAZAR can reduce its employees’ physical risks. HANKAMP has also gained a better understanding of the aspects which need attention, when designing/utilizing a robot. Specifically, managing the workers’ expectations and the way they perceive the robot is paramount. To this end, in the near future, HANKAMP will focus on how to set the right expectations and how to help workers gain a better understanding of robots, to intrinsically motivate their usage. Otherwise, this type of solution may end up wasted—i.e., never used by workers—as it occurs with simpler, yet similar, existing in-house solutions (e.g., overhead cranes).
